# Minimum important difference is minimally important in sample size calculations

**DOI:** 10.1186/s13063-023-07092-8

**Published:** 2023-01-17

**Authors:** Hubert Wong

**Affiliations:** School of Population & Public Health, University of British Columbia, 2206 East Mall, Vancouver, BC V6T 1Z3 Canada

**Keywords:** Clinical trial, Power, Effect size, Assumed benefit

## Abstract

Performing a sample size calculation for a randomized controlled trial requires specifying an assumed benefit (that is, the mean improvement in outcomes due to the intervention) and a target power. There is a widespread belief that judgments about the minimum important difference should be used when setting the assumed benefit and thus the sample size. This belief is misguided — when the purpose of the trial is to test the null hypothesis of no treatment benefit, the only role that the minimum important difference should be given is in determining whether the sample size should be zero, that is, whether the trial should be conducted at all.

The true power of the trial depends on the true benefit, so the calculated sample size will result in a true power close to the target power used in the calculation only if the assumed benefit is close to the true benefit. Hence, the assumed benefit should be set to a value that is considered a realistic estimate of the true benefit. If a trial designed using a realistic value for the assumed benefit is unlikely to demonstrate that a meaningful benefit exists, the trial should not be conducted. Any attempt to reconcile discrepancies between the realistic estimate of benefit and the minimum important difference when setting the assumed benefit merely conflates a valid sample size calculation with one based on faulty inputs and leads to a true power that fails to match the target power.

When calculating sample size, trial designers should focus efforts on determining reasonable estimates of the true benefit, not on what magnitude of benefit is judged important.

## Background

When calculating the required sample size for a definitive randomized controlled trial, an assumed benefit (that is, the mean improvement in outcomes due to the intervention) must be specified. In broad terms, the main approaches to setting the assumed benefit include (1) select a value for Cohen’s (standardized) effect size, (2) use an estimate obtained from previous studies (possibly pilot studies), (3) elicit a value from experts, (4) use a value that is considered a realistic benefit, and (5) use a value that represents the minimum important difference (MID). What constitutes a MID often is not well-defined, but criteria typically are anchored to the notions of the smallest benefit that would be of interest to stakeholders or the benefit that would need to be observed to justify a change in practice. The minimum clinically important difference (MCID) (or simply clinically important difference (CID), when the connection with a minimum value is implied) often is used synonymously with the MID. This commentary does not summarize the extensive literature discussing the advantages and shortcomings of the various approaches, but instead focuses on the (mis-)use of the MID and the central role of the realistic benefit. Unless noted otherwise, it is assumed throughout that the null hypothesis being tested is that the true benefit is zero.

The DELTA^2^ study [1] (along with its precursor DELTA [2]) represents perhaps the most comprehensive investigation into how the assumed benefit, called the “target difference” in that study, should be set. DELTA^2^, commissioned by the UK’s Medical Research Council, comprised literature reviews, a Delphi process engaging stakeholders, a 2-day workshop, and a finalization of core guidance. DELTA^2^ concluded that “The target difference between treatments that is considered realistic or important by one or more key stakeholder groups plays a critical part in the sample size calculation.” However, no specific guidance was given on how one should reconcile potential discrepancies between “realistic” and “important” values, beyond acknowledging (1) that “The target difference does not necessarily have to be the minimum value that would be considered important if a larger difference is considered a realistic possibility or would be necessary to alter practice” and (2) that others have argued that the target difference should satisfy both criteria [3]. In current practice, sample size justifications often rely on the “important” aspect—among the 30 most recent protocols (in which a definitive sample size calculation was warranted) published in *Trials* leading up to July 18, 2022, we found that five protocols (17%) reported setting the assumed benefit to a MID value (referred to as either a MCID or a CID). Acceptance of the use of MCID or CID is so widespread that literature and software illustrating sample size calculation commonly instruct users to enter a MCID or CID for the assumed benefit with little or no discussion of the appropriateness of doing so. The manner in which MID currently is used in determining sample size is misguided and leads to incorrect justifications of the required sample size.

## What is wrong with using the MID?

The primary motivation for considering the MID when setting the assumed benefit is the notion that this will help to ensure that the trial can determine whether a meaningfully important benefit exists. Chuang-Stein et al. [4] discussed three different ways to calculate sample size based on the MID according to the goal of the trial: (1) showing the effect is statistically significant (i.e., > 0) when the true benefit is equal to the MID, (2) showing the effect is both statistically significant and at least as large as the MCID, and (3) showing the effect is statistically larger than the MCID (i.e., the null hypothesis is that true benefit is equal to the MID). The first goal reflects current practice; yet straightforward calculations (or more simply, a well-constructed graph) show this approach does not answer whether an important difference exists. For a two-sample *Z*-test with a target power of 80% and a two-sided alpha of 0.05, (1) a positive trial result only guarantees that the *point estimate* of the true benefit is at least 0.7 × MID; (2) the probability that the *point estimate* achieves the MID is 50%; (3) the probability of “proving” the true benefit is at least the MID (i.e., obtaining a *95% confidence interval* with a lower bound above the MID) is 2.5%! Thus, setting the assumed benefit to the MID clearly is inadequate for generating strong evidence for a MID benefit. When the outcome is a continuous variable, an additional challenge is connecting mean improvement to the proportion of individuals who experience a benefit greater than the MID. Resources such as the PROMID repository (www.promid.org) are available to assist researchers with this challenge.

However, there is a more fundamental problem than choosing the wrong numerical value for the assumed benefit based on the MID. Although the primary aim of the sample size calculation is to ensure that the trial will generate sufficient information, a fundamental requirement is that the calculation must be *valid*. That is, adopting the calculated sample size would result in a trial whose true power (nearly) equals the target power used in the calculation. Assuming other inputs are accurate, this occurs when the value for the assumed benefit is close to the true benefit; when the assumed benefit is far from the true benefit, the calculated sample size simply is meaningless. Thus, the appropriate choice for the assumed benefit is a value that is judged to be a realistic estimate for the true benefit. What magnitude of benefit we consider to be important or hope for has no impact on whether the true power achieves the target power.

For example, suppose we set the sample size based on an assumed benefit to 4 units, reflecting our best estimate for the true benefit. If indeed, the true benefit is near 4 units, then the true power of the trial will match the target power. Now suppose that a 1-unit benefit is considered important. We should be satisfied that our trial likely will yield reasonably strong evidence that the intervention provides an important benefit and proceed using this sample size. We should not change the assumed effect to 1 unit; that would increase the sample size by a factor of 16 and result in an overpowered (and potentially a too-large-to-be-feasible) trial. Conversely, suppose that the minimum important benefit is 8 units. We should acknowledge that the trial is highly unlikely to show that the intervention produces a meaningful benefit and simply abandon the trial. We should not change the assumed effect size to 8 units; this would decrease the calculated sample size by a factor of 4 and result in an underpowered trial that even more surely will not show a meaningful benefit. Any attempt to reconcile discrepancies in judgments between what is important and what is realistic (e.g., by taking the minimum or an in-between value) to obtain a value for the assumed benefit conflates the goal of ensuring sufficient information with the requirement that the calculation be valid and leads to a sample size that addresses neither. This argument does not imply that only one value for the realistic benefit should be considered, given the uncertainty about the true benefit, but judgments about what is an important benefit should not be used to inform judgments about what is a realistic benefit.

## How should the realistic benefit be determined?

As summarized in the DELTA^2^ guidance, methods that could be used to inform what is a realistic benefit include pilot studies, opinion seeking, and review of the evidence base. Cautions have been raised regarding the uncritical acceptance of each of these methods. Because the estimated treatment effect in a pilot study is highly uncertain, multiple authors [5, 6] have argued that using this value for the assumed effect would lead to a high risk of either inadequate power (if the true effect was overestimated) or to incorrectly concluding that the planned trial is infeasible or not justifiable (if the true effect was underestimated). Others [7] have expressed concern that trial designers typically have an inherent optimism about the intervention’s efficacy, which could lead them to overestimate the true benefit and lead to inadequate sample size and power. Results from reviewing the evidence base may not be transportable to the planned trial due to differences in the precise specifications of the intervention and its delivery, the study populations, and the settings.

All of the foregoing concerns are valid, but ultimately, a value for the realistic effect must be chosen. This judgment should be made through thoughtful consideration of the trial context and all available evidence—information should not be discarded simply because it is not “ideal.” For example, the risks of adopting a treatment effect estimate from a pilot study because it has large uncertainty notwithstanding, it would be counterproductive to ignore this information altogether. Because different sources, types, and amounts of information may need to be considered, each with uncertain levels of relevance, developing a universally applicable synthesis process may not be practical. However, identifying such a process is not as important as ensuring that whatever process is undertaken, it considers the task thoroughly and is reported transparently (as advocated in the DELTA^2^ guidance) to facilitate assessing the degree of support for the chosen assumed benefit. Recent practice falls far short of this goal as evidenced in the 30 *Trials* protocols referenced above. Sixteen protocols stated the choice of the assumed benefit was obtained from/based on previous studies/literature, yet only two provided any discussion of why or the extent to which the results from previous studies ought to be applicable to the planned trial, despite differences in study populations, intervention, design, or outcome definitions. More disappointing, eight protocols stated the assumed benefit without providing any indication of how the value was chosen. One protocol did not perform a sample size calculation on the grounds that there was no existing data to inform the assumed benefit. The poor adherence to the DELTA^2^ guidance may be due to some of these trials having been designed prior to the publication of the guidance or to lack of awareness of its publication.

It is not reasonable to expect that trial designers are able to choose with confidence a single value for the assumed benefit, so a range of values should be considered. But again, for the results to be meaningful, this range must reflect realistic values, not what values are considered important. When insufficient evidence is available to judge what would be a realistic assumed benefit, an adaptive trial design incorporating sample size re-estimation would be appropriate, rather than attempting to fix a sample size based on an assumed benefit which may be far from the truth.

## Conclusion

When the trial’s null hypothesis is no treatment benefit, the best way to ensure the true trial power matches the target power is by setting the assumed benefit to a realistic estimate of the true benefit in the sample size calculation. Attempting to adjust that sample size by taking the minimum important difference into consideration merely leads to an invalid sample size calculation. The minimum important difference should play no role in setting the sample size; however, to provide support for conducting the trial, trialists should report on the minimum important difference and provide the rationale for why the realistic benefit is expected to be (substantially) greater than the minimum important difference.

## Data Availability

Not applicable

## Abbreviations

CID: Clinically important difference
MCID: Minimum clinically important difference
MID: Minimum important difference
